# Assessing the effectiveness of a pharmacist-delivered smoking cessation program in the State of Qatar: study protocol for a randomized controlled trial

**DOI:** 10.1186/s13063-015-0570-z

**Published:** 2015-02-26

**Authors:** Maguy Saffouh El Hajj, Nadir Kheir, Ahmad Mohd Al Mulla, Daoud Al-Badriyeh, Ahmad Al Kaddour, Ziyad R Mahfoud, Mohammad Salehi, Nadia Fanous

**Affiliations:** College of Pharmacy, Qatar University, Doha, POBOX 2713, Qatar; Smoking Cessation Clinic, Medicine Department, Hamad Medical Corporation, Doha, Qatar; Department of Health Care Policy and Research, Weill Cornell Medical College, New York, NY USA; Department of Global and Public Health, Weill Cornell Medical College, Doha, Qatar; Department of Mathematics, Statistics and Physics, College of Arts and Sciences, Qatar University, Doha, POBOX 2713, Qatar

**Keywords:** Qatar, Tobacco control, Smoking cessation, Pharmacist

## Abstract

**Background:**

It had been reported that up to 37% of the adult male population smokes cigarettes in Qatar. The Global Youth Tobacco Survey also stated that 13.4% of male school students aged 13 to 15 years in Qatar smoke cigarettes. Smoking cessation is key to reducing smoking-related diseases and deaths. Healthcare providers are in an ideal position to encourage smoking cessation. Pharmacists are the most accessible healthcare providers and are uniquely situated to initiate behavior change among patients. Many studies have shown that pharmacists can be successful in helping patients quit smoking. Studies demonstrating the effectiveness of pharmacist-delivered smoking cessation programs are lacking in Qatar. This proposal aims to test the effect of a structured smoking cessation program delivered by trained ambulatory pharmacists in Qatar.

**Methods/Design:**

A prospective, randomized, controlled trial is conducted at eight ambulatory pharmacies in Qatar. Participants are randomly assigned to receive an at least four-session face-to-face structured patient-specific smoking cessation program conducted by the pharmacist or 5 to 10 min of unstructured brief smoking cessation advice (emulating current practice) given by the pharmacist. Both groups are offered nicotine replacement therapy if feasible. The primary outcome of smoking cessation will be confirmed by an exhaled carbon monoxide test at 12 months. Secondary outcomes constitute quality-of-life adjustment as well as cost analysis of program resources consumed, including per case and patient outcome.

**Discussion:**

If proven to be effective, this smoking cessation program will be considered as a model that Qatar and the region can apply to decrease the smoking burden.

**Trial registration:**

Clinical Trials NCT02123329.

## Background

The leading preventable cause of ill health today is cigarette smoking [1]. It is a major risk factor for several causes of death including heart disease, chronic lung diseases and lung cancer [2]. It kills one in ten adults globally and causes more than 5 million deaths annually. If present trends continue, the number of smoking-related deaths is expected to reach more than 8 million deaths per year by 2030 [3]. The adverse health consequences of tobacco use not only compromise smokers’ quality of life, but also entail significant direct and indirect economic costs on society. The 2008 World Health Organization (WHO) report states that the global healthcare costs attributable to tobacco use reach hundreds of billions of US dollars each year [4]. Smoking cessation is key to reducing the number of smoking-related diseases and deaths. At any age, quitting smoking improves quality of life (QoL) and confers considerable health benefits including reduced risk of coronary heart disease, stroke and smoking-related cancers [5,6]. The World Bank suggests that if adult tobacco consumption were to decrease by 50% by the year 2020, approximately 180 million tobacco-related deaths could be avoided between now and 2050 [7]. Health professionals are in an ideal position to encourage smoking cessation. A short intervention by them can considerably increase the smoking cessation rate [8]. Of all health professionals, pharmacists are the most easily accessed by the public and are uniquely situated to initiate behavior change among smokers [9]. They can provide education and advice without prior appointment and with no direct or additional cost to individuals [10]. They regularly interact with patients obtaining medications for smoking-related diseases. In addition, the increased availability of pharmacologic smoking cessation aids including some formulations of nicotine replacement therapy over the counter in pharmacies in many countries gives the pharmacists a distinct opportunity to serve as front-line health providers who can play a major smoking cessation role [11]. Moreover, available data support the provision of smoking cessation services in ambulatory and community pharmacies. Many studies have shown that pharmacists can be effective providers of tobacco cessation interventions and can be successful in helping patients quit smoking [12-20] and that these pharmacy interventions can be cost-effective or cost-beneficial [21-24]. Additionally, in many countries there is extensive support at the professional level for the involvement of pharmacists in smoking cessation. The Canadian Pharmacists Association developed a Joint Statement on Smoking Cessation. This statement promotes the involvement of health professionals including pharmacists in tobacco prevention and cessation [8]. The International Pharmaceutical Federation (FIP) issued a statement of policy on the role of pharmacists in promoting a tobacco-free future [25]. The European Forum adopted a pharmacists’ charter on action against smoking, which states that pharmacists should consider smoking cessation as an essential element of their professional responsibilities [26]. The US Public Health Service published evidence-based clinical guidelines recommending that all health care providers, including pharmacists, should offer smoking-cessation interventions [27]. The American Society of Health-System Pharmacists’ (ASHP) Therapeutic Position Statement on the Cessation of Tobacco Use strongly encourages pharmacists as well as other healthcare providers to integrate identification of tobacco users and delivery of tobacco-cessation interventions into their routine patient care. As per the ASHP statement, these interventions should address five key elements of comprehensive tobacco cessation counseling (the 5 As): (1) asking patients about tobacco use, (2) advising users to quit, (3) assessing their readiness to quit, (4) assisting them with quitting using behavior modification and smoking cessation medications, and (5) arranging follow-up care. If comprehensive tobacco-cessation counseling cannot be provided because of time constraints or practice site logistics, pharmacists can use a truncated 5 A model whereby they ask about tobacco use, advise tobacco users to quit, and then refer patients to smoking cessation providers or programs [28].

In Qatar, tobacco use is a major public health problem that, in addition to the associated morbidity and mortality, constitutes a huge burden on healthcare services. To control tobacco use, Qatar has adopted the international approaches recommended by the World Health Organization (WHO) and regional plan of Arab countries in the Gulf. These approaches are based on three strategies: (1) imposing legislative tobacco control measures, for example, banning smoking in enclosed public places and prohibiting selling cigarettes, tobacco or its derivatives to anyone less than 18 years old, (2) providing smoking cessation services for free in two hospital-based smoking cessation clinics and (3) implementing antismoking public awareness activities [29]. Despite these approaches, in Qatar up to 37% of adult males currently smoke tobacco [30] and 13.4% of male school students aged 13 to 15 years smoke cigarettes according to the 2007 Global Youth Tobacco Survey [31]. Furthermore, smoking-related diseases are the most prevalent in Qatar. Ischemic heart disease, as an example, was ranked as one of the leading causes of burden of disease in Qatar in 2010 [32], and Qatar has a higher age standardized lung cancer incidence rate than many countries in the Gulf (8.95 per 100,000) [33]. In addition, according to a recent survey done by Weill Cornell Medical College in Qatar, at least 1 billion cigarettes are smoked in Qatar each year, averaging 12,000 per individual, and 65 million US dollars (USD) are spent on cigarettes annually; 150 million USD are used to cover the healthcare costs of patients affected by smoking-related diseases [34]. The potential for pharmacist-assisted tobacco cessation is therefore huge and could have a considerable impact on smoking rates, prevention of tobacco-related diseases and enhancement of public health in Qatar. Around 700 registered pharmacists practice in public and private ambulatory care clinics and in community pharmacies in Qatar. If each of these pharmacists successfully assists just one tobacco user in quitting each month, this would result in more than 8500 quitters annually. However, according to a study conducted by Qatar University’s College of Pharmacy and funded by the Qatar Foundation under its Undergraduate Research Experience Program (UREP), Qatar’s ambulatory and community pharmacists are not fully engaged in smoking cessation activities. Eighty percent of Qatar’s ambulatory and community pharmacists rarely, never or sometimes ask their patients about their smoking status, and 53% rarely, never or sometimes offer smoking cessation counseling to nicotine therapy purchasers. When meeting patients who smoke, only 45% of pharmacists discuss the adverse health effects of smoking, 41% assist them in quitting and 16% arrange follow-up sessions with them to assess their progress in quitting smoking. However, more than 85% of pharmacists are interested in providing smoking cessation counseling and believe that it is an important activity [35]. The pharmacists’ perceptions regarding the importance of smoking cessation can be transformed into action if a smoking cessation model is provided that these pharmacists can use in routine practice. Unfortunately, an intensive, patient-specific program of smoking cessation designed exclusively for implementation in community and ambulatory pharmacies is not available in Qatar. In addition, evidence regarding the effect of such a program is lacking in Qatar.

Also important, in addition to the numerous knowledge gaps identified above in relation to Qatar, there are also no data on how cost beneficial the running of smoking cessation programs like the currently proposed one is, added to a lacking data on the associated resource implications in the pharmacy setting from the Qatari perspective.

### Study objectives

This proposal describes a study that is currently being implemented in eight public and private ambulatory clinic pharmacies in Qatar. Our hypothesis is that a patient-specific structured smoking cessation program delivered by smoking cessation-trained ambulatory pharmacists would be significantly more effective in helping smokers stop smoking and achieving long-term smoking abstinence than brief unstructured pharmacist-delivered advice.

We also hypothesize that quitting smoking would lead to significant improvement in health-related quality of life.

The study objectives are:

- Primary: To test the effect of a face-to-face structured patient-specific smoking cessation program delivered by trained ambulatory pharmacists on smoking cessation rates in Qatar
- Secondary: To assess the change in health-related quality of life in quitters vs. non-quitters
- Secondary: To explore the resource utilization of implementing the smoking cessation program as delivered by the ambulatory pharmacists in Qatar. This is a cost analysis of the overall cost of running the proposed patient-specific cessation program, including cost-effectiveness analyses.

## Methods/design

### Study design

The study is a prospective randomized controlled trial comparing the effectiveness of a face-to-face structured patient-specific smoking cessation program conducted by trained ambulatory pharmacists versus brief unstructured pharmacist-delivered advice on smoking cessation rates.

### Study sites

The study is implemented in eight public and private ambulatory pharmacies in the State of Qatar. These pharmacies are located within large ambulatory clinics that serve a large proportion of the Qatar population. To prevent cross contamination with the study, none of these clinics has established smoking cessation programs. Each study pharmacy is staffed by at least three pharmacists. Two pharmacists from each pharmacy are invited to participate in the study. The study is taking place in a semiprivate area in the pharmacy. This area is secluded enough to ensure adequate patient confidentiality.

### Study pharmacists’ training

Prior to starting the study, each study site pharmacist was sent a copy of the study methodology along with a written literature review on smoking cessation and asked to study the material.

Two to three weeks after receipt of the material, the study pharmacist attended a 2-day (8 h/day) smoking cessation training workshop that was organized by the Hamad Medical Corporation (HMC) smoking cessation clinic team and Qatar University (QU) College of Pharmacy.

The training team consisted of Dr. Ahmed al Mulla (Public Health and Disease Control Consultant and HMC smoking cessation clinic head), Dr. Maguy El Hajj (Study Principal Investigator/Assistant Professor and CPP Chair at QU College of Pharmacy), Dr. Nadir Kheir (Associate Professor and CPPD coordinator at QU College of Pharmacy) and Dr. Mohamad Haniki Nik Mohamed [Deputy Dean (Academic Affairs) in Kulliyyahof Pharmacy at the International Islamic University Malaysia (IIUM)]. Dr. Haniki is considered an international authority in the area of substance abuse, including cigarette smoking.

The HMC smoking cessation clinic is one of the smoking cessation facilities in Qatar. The clinic is run by highly qualified smoking cessation consultants and has a smoking cessation success rate of 40%. In addition to offering smoking cessation treatment, this clinic contributes to community education on the dangers of smoking among the citizens and residents of Qatar and provides smoking-related lectures and seminars for all age groups in the state [36].

The general objective of this workshop is to provide the study pharmacists with enough smoking cessation knowledge and the necessary skills and tools to assess and treat smokers. A smoking cessation training manual that was developed by the study training team is extensively used in the workshop. This manual is also used as an on-site resource by the study pharmacists in their smoking cessation program.

The workshop covered the following: smoking epidemiology and risks, benefits of quitting, a transtheoretical model for behavior change as applied to smoking cessation, the necessary elements for documentation in the patient smoking cessation profile, behavioral modification techniques, classification of smokers according to their stage of change (5 stages: precontemplation, contemplation, preparation, action and maintenance), nicotine replacement therapy (NRT: nicotine patch, lozenge and gum), patient counseling techniques, development of a personalized action plan, use of the exhaled carbon monoxide test, detailed instructions on the program methodology and other needed elements.

In addition, the pharmacists’ training workshop included training the pharmacists on how to follow the study protocol exactly. The study team walked the pharmacists through the study protocol including how to randomly allocate participants to the intervention and control groups, how to deliver the intervention and how to deal with control patients.

During the training workshops, emphasis was also placed on the importance of behavioral and lifestyle modifications. Pharmacists were instructed to encourage smokers who are anticipating quitting to identify the situations that may increase their risk of smoking such as having smokers within the household or at the workplace as well as getting into stressful situations. After identifying these situations, the pharmacists were provided with information they could give to smokers about strategies to improve coping including lifestyle changes to decrease stress and improve QoL, removing visual cues for smoking and staying away from these situations that normally make patients want to smoke [37]. In addition, the pharmacists were trained to provide the smokers with information about what to expect during quit attempts and to address any individual concerns that the smokers may have [38]. Furthermore, they were advised to encourage the smokers to notify family and friends of the plan to quit and to ask for their support [37].

Participant counseling and interviewing techniques were also part of the training workshops. To enhance learning, the pharmacists applied these techniques using patient role-plays in small groups. Topics that were reviewed included counseling skills, verbal and nonverbal communication skills including use of empathy and active listening, use of open-ended and priming questions, verification of patient understanding, overcoming of communication barriers, communication with difficult patients, communication with patients not interested in discussing smoking cessation and use of strategies to improve medication adherence.

Documentation was an important element of the workshop. The pharmacists were trained to document the patient’s smoking, medical and medication history, basic demographic characteristics, staging, assessment, education, counseling, follow-up and the personalized action plan to quit smoking. In addition, they were instructed on how to fill in the Fagerstrom test for nicotine dependence (FTND). The FTND is a six-item questionnaire designed to assist the healthcare professional in determining a patient’s degree of nicotine dependence [39]. This questionnaire has already been used in smoking-related research studies in the Middle East area [40,41]. Furthermore, they were instructed to measure the smoker’s health-related QoL using the Smoking Cessation Quality of Life (SCQOL) questionnaire. This instrument was developed and validated for use in smoking cessation [42,43] and has already been used in previous studies that tested the effectiveness of a pharmacist-delivered smoking cessation program [16]. This questionnaire tackles a total of 13 general and smoking cessation-specific quality-of-life domains including physical functioning, role-physical, bodily pain, general health, vitality, social functioning, role-emotional, mental health, social interactions, self-control, sleep, cognitive functioning and anxiety [43].

### Study/program marketing

The study pharmacists were trained to recognize many opportunities for the promotion of the smoking cessation program as possible. For example, when dispensing prescriptions, the study pharmacists were encouraged to ask patients about smoking and to tell them about the smoking cessation program. In addition, study site pharmacists displayed posters and leaflets in their pharmacy describing the smoking cessation program. Furthermore, the general practitioners and dentists practicing in the study ambulatory clinics were sent letters inviting them to refer their patients who are interested in quitting to the study pharmacies.

### Screening and eligibility criteria

Interested smokers meet with the study pharmacists. The study pharmacists use a scripted questionnaire to screen smokers in order to assess their eligibility for the study. Eligible participants are patients aged 18 years and older who currently smoke one or more cigarettes daily for 7 days, are motivated to quit, i.e., in the preparation stage of the stage-of-change model, able to communicate in Arabic or English, and are willing to and capable of attending the scheduled sessions at the study pharmacies. The motivation to quit is determined by the pharmacist using the transtheoretical model of behavior change [44]. Smokers in the precontemplation or contemplation stages are advised to quit, are provided with educational materials regarding tobacco cessation and the health benefits of quitting, and are encouraged to come back to the pharmacy when they feel they are ready to commit to quitting smoking.

Exclusion criteria are (1) use of other nicotine or tobacco products and (2) current use or use in the last 30 days of smoking cessation aids or medications, (3) planning to leave Qatar in the next 12 months, (4) presence of any major medical condition that would prevent use of the nicotine replacement therapy including hypersensitivity to the products, history of or recent myocardial infarction, life-threatening arrhythmias, severe or worsening angina, uncontrolled hypertension and temporomandibular joint disease (in case of nicotine gum), (5) pregnancy and (6) psychiatric illness or another debilitating condition that would interfere with participation in the study.

### Study enrollment

Following the completion of the screening procedure, the study pharmacist gives the eligible participant pertinent background information on the program, explaining what his or her participation would involve (including potential benefits, risks, inconveniences, discomforts), and his or her right to confidentiality. In order to enroll in the study, the interested participant is asked to sign an approved consent form. Once the consent form is signed, the pharmacist collects the participant’s contact information and sets an appointment with him or her at a date and time that is suitable for both of them.

### Initial appointment and randomization

One to two days before his or her scheduled appointment, the study pharmacist contacts the enrolled participant to remind him or her about the appointment. At this initial appointment, which takes around 30 min, the study pharmacist first collects the participant’s sociodemographic characteristics, current medical problems and medications, smoking history (i.e., number of cigarettes per day, duration of smoking, previous quit attempts, smoking habits of other family members and the participant’s reasons for wanting to quit smoking) and vital signs (i.e., blood pressure and pulse). In addition, the pharmacist assesses the smoker’s level of nicotine dependence using the Fagerstrom Test for Nicotine Dependence and measures his or her baseline exhaled carbon monoxide (CO) level using the exhaled CO test. Furthermore, the pharmacist administers the smoking cessation quality-of-life (SCQOL) questionnaires. After baseline data collection, the smoker is randomly assigned to either the intervention group or the control group. Randomization is already anonymously preset by the study statistician for each study pharmacist. According to the participant number and the preset list, the participant will be randomized to the intervention or control group. A new appointment is set with the participants assigned to the intervention group. Participants assigned to the control group receive brief unstructured smoking cessation advice as outlined below. Participants in both groups do not pay for their participation.

### Intervention group

Participants assigned to the treatment group participate in a face-to-face four-session program at the pharmacy delivered by the study pharmacist at 2-4-week intervals over 8 weeks. The sessions are set at a date and time that are convenient for both the pharmacist and participant.

#### First session

The first session is time-intensive, taking around 30 min. In this session, the study pharmacist facilitates the participant’s preparation to quit. The participant selects a quit date within the next 2-4 weeks. Based on the transtheoretical model of change, this would give him or her time to get prepared to enter the action stage on the quit date.

The pharmacist discusses the benefits of smoking cessation with the participant and provides him or her with tailored behavioral and lifestyle strategies. These strategies take into consideration the participant behaviors, values and beliefs. For example, if the smoker states that the hardest time would be at work when others are taking breaks to smoke, the pharmacist may suggest ways to spend the time during breaks. As outlined under the training section, the pharmacist also provides the participant with information about what to expect during the quit attempt with suggestions to cope with the early days of quitting. In addition, the pharmacist encourages the participant to seek family support during the attempt.

To prevent nicotine withdrawal symptoms, the participant is also offered, without cost, nicotine replacement therapy (NRT) as a patch or lozenge depending on his or her previous experience, preference and adverse effect profile. Other NRT dosage forms, e.g., an inhaler, nasal spray and sublingual tablets, are not available in Qatar. The gum is usually not tolerated by patients. The participant is not offered the other smoking cessation aids, including bupropion and varenicline, as they require a prescription in order to be dispensed in Qatar. Before dispensing the NRT, the study pharmacist ensures that the participant does not have any contraindications to nicotine therapy and is not taking any medication that would interact with NRT. The NRT is started on the participant’s intended quit date. Patient counseling about NRT is provided to each participant regarding the dosage, administration, scheduling, duration of therapy, adverse effects, interactions and proper disposal.

Participants who take the nicotine patch start at 21 mg if they smoke ten cigarettes or more per day. Otherwise, they receive a 14-mg patch. Both patches are continued for 6 weeks before being tapered. The 21-mg patch is decreased to 14 mg, continued for 2 weeks and decreased to 7 mg for a final 2 weeks. Patients who start with the 14-mg patch are tapered to 7 mg for a final 2 weeks [45]. Participants who take the nicotine lozenge use the 1-mg pieces. It is continued for 6 weeks as one lozenge every 1-2 h before being tapered. Then the participant takes one lozenge every 2-4 h for weeks 7 to 9, then one lozenge every 4-8 h for week 10-12 [46].

A personalized action plan is created by the pharmacist to document the participant’s quit date, nicotine replacement therapy dosage schedule and counseling, suggested behavioral and lifestyle strategies, and date/time of the next appointment. This plan is signed by both the participant and pharmacist.

#### Follow-up sessions

The first follow-up session is scheduled 1 week after of the participant’s quit day and takes around 20 min. In this session, the pharmacist determines the participant’s smoking status, measures his or her blood pressure/pulse/exhaled CO level, and assesses the NRT tolerability. If the patient succeeds in quitting, the pharmacist provides reinforcement and addresses problems associated with quitting such as managing withdrawal symptoms, coping with cravings, relieving stress, and managing fear of weight gain and of change in relationships with other smoking friends or relatives. If the participant fails to quit smoking, the pharmacist carefully reviews the participant’s experience during the attempt to quit and works through the identified problems. If the participant does not tolerate the NRT side effects (e.g., his or her blood pressure or heart rate increases), the pharmacist asks the participant to stop the NRT and refers him or her to the HMC smoking cessation clinic for further smoking cessation support. This participant is no longer enrolled in the study.

**Figure 1:**
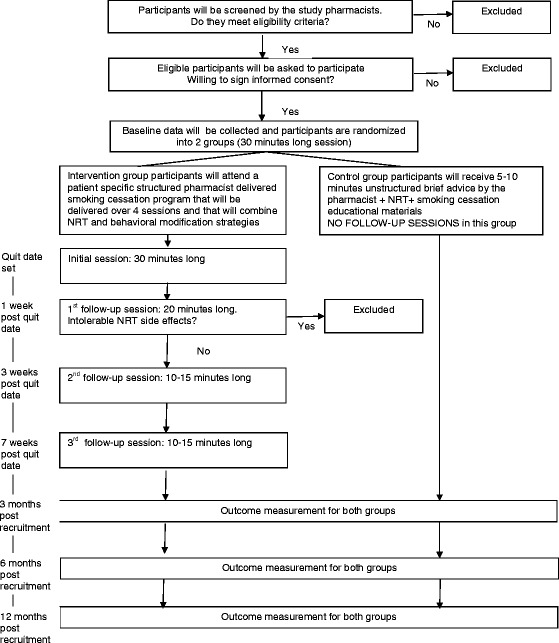
**Participant flow during the study.**

The second follow-up session is scheduled 2 weeks after the first follow-up session. The third follow-up session is set at 4 weeks after the second follow-up session. These sessions take no more than 10 or 15 min each. In these sessions the pharmacist assesses the participant’s smoking status, blood pressure/heart rate/exhaled CO level and NRT tolerability and provides NRT refills. In addition, he or she focuses on maintenance of smoking abstinence with cognitive-behavioral strategies to prevent relapse. The pharmacist documents any action taken by him/her at each follow-up session. After the third follow-up session, all participants are instructed to call or visit the pharmacy for questions or to receive additional support as needed. For more information on participant flow during the study, see Figure 1.

#### Control group

Participants in the control group receive 5-10 min of unstructured brief smoking cessation counseling by the pharmacist (emulating current practice). In addition, they are provided with educational materials about smoking cessation and are offered nicotine replacement therapy (NRT). Before dispensing the NRT, the study pharmacist ensures that the participant does not have any contraindications to nicotine therapy and is not taking any medication that would interact with NRT. The choice of the NRT dosage form (patch or lozenge) depends on the participant’s previous experience, preference and adverse effect profile. The NRT dosage schedule and duration of therapy are similar to those in the intervention group. Control participants are not asked to attend any follow-up sessions.

The education materials address the following topics: the adverse effects of smoking, benefits of smoking cessation, the methods of quitting, setting the quit date, coping with nicotine withdrawal symptoms, getting emotional and social support, and monitoring the blood pressure and pulse when using NRT and avoiding relapse.

The study team invests a good deal of time orienting the study pharmacists on their role and training them on how to deal with the control and intervention groups. Part of that orientation is on how ‘not’ to provide any intervention to the control subset of smokers.

### Study piloting

Study pharmacists were provided with assistance in setting up the study pharmacies and given some time to familiarize themselves with the study protocol prior to commencement of the study. Then each pharmacist was asked to pilot the study in the presence of the study team. Piloting occurred by screening/recruiting around four patients in each site and by implementing the study protocol. Feedback was provided as required to the study pharmacists on the quality of patient counseling, patient education and documentation. Very minor adjustments or refinements were made to the study protocol and study tools including the patient consent form, Fagerstrom test for nicotine dependence (FTND) and smoking cessation quality-of-life (SCQOL) questionnaire as needed.

### Continuous quality improvement and evaluation plan

At all stages of the study implementation, the study pharmacists are closely supervised by the study team. Supervision is through several mechanisms including meetings, communication and site visits.

#### Meetings

Regular meetings are invaluable for high-quality supervision. Meetings are used to guide the project toward its objectives through team involvement. Bi- or triweekly meetings involving all the team members including the study pharmacists are held. In these meetings, study harmacists summarize their activities during the previous 2-3 weeks, and the study team offers them constructive feedback, acknowledges their input and suggests new directions to them. In addition, ad-hoc meetings are conducted between the study pharmacists and the study team where necessary to address any irregular issues that might arise.

#### Communication

The study team members are in direct touch with each other and with the study pharmacists through electronic communication such as emails.

#### Site visits

To maintain quality control in the smoking cessation study pharmacies, the study team visits each study pharmacy at least once every 2 to 3 weeks to monitor data collection, check protocol adherence, ensure the consistency of program delivery, address any queries that the study pharmacists might have in implementing the study, review patients’ folders for appropriate documentation, provide any on-site support required and provide individual pharmacists with feedback on their performance.

Supervision is crucial to:

- Orient study pharmacists to the study’s objectives and methodology and to their role in the study
- Monitor and follow-up the progress of the study
- Tackle any issues or concerns that might arise with the study pharmacists while the study is executed
- Share study updates
- Motivate and support the study pharmacists
- Ensure adherence to the study protocol in the different study sites
- Ensure standardization of study implementation in the different study sites

### Overview of utilization cost

A comparative overview of the cost of resource utilization (economic analysis) will be undertaken for the study arms in this study.

#### Model inputs and cost determination

This pharmacoeconomic analysis will be based on extrapolating data on resources from the randomized controlled trial; these data will be collected prospectively. Prospective data collection enhances the accuracy and robustness of data and limits bias.

The economic analysis is performed from a social perspective. Direct medical costs will be evaluated to the best available, including in relation to NRT, CO monitoring tests and salaries. Direct nonmedical costs will also be evaluated and will include participants’ travel costs to pharmacies (e.g., taxi) and the paperwork and forms. The indirect costs will be accounted for as well, in relation to lost productivity (if any), whether for participants or any unpaid caregivers that participants utilize. Important is that any protocol-driven costs (e.g., researchers salaries) will be excluded from the analysis.

The monetary value of resources will be collected from internal sources at the Qatari clinics. It is worth noting that costs involved in this study will be based on wholesale prices, as paid by clinics.

All calculated costs will be in Qatari Riyal, adjusted for the financial year 2015-2016, utilizing the Qatari Health Consumer Price Index as appropriate, and no discounts will be applied given the short timeframe of the analysis.

The economic evaluation will be undertaken via decision analytic modeling, where a decision tree will be structured to follow the different options and consequences in the management pathways. For each of the participants, the proposed model may include five possible treatment outcomes depending on whether participants quit smoking, and if they do, after what duration. Smokers will be initially assigned to one of two pathways depending on whether they quit smoking at enrollment. Smokers who do not quit at enrollment will be further followed until assigned to one of two pathways, depending on whether the smoker quits smoking after 3 months or not. If not, smokers will be followed up until further assigned to one of two pathways, based on whether they quit smoking after 6 months or not. Those who do not quit by then will be followed up until assigned to one of two pathways depending on whether they quit at 12 months of follow-up or not, which will be the end of follow-up in any case. Smokers who quit at enrollment or after 3, 6 or 12 months will each be followed up separately until the end of the study. Based on this, in addition to data on resources consumed and their monetary values, the model will generate a weighted average cost for patient cessation and quality-of-life adjustment. As per the general decision analysis principles, this will be calculated as the sum-product of the costs of the management outcomes and the respective probabilities.

The costs of managing the single episode of smoking cessation will be compared among different management options and against budgeted costs of clinics/government institutions.

#### Sensitivity analysis

The goal of sensitivity analysis is to indicate the robustness of the evaluation conclusion against any potential variations in the model inputs. Deterministic and probabilistic sensitivity tests will be produced by modifications on the base case values of several key variables, in relevance to costs and probabilities, to evaluate the robustness of the study economic outcome. Base case values will be substituted by the highest and lowest values within a reasonable range of values. Where a substitution changes the study economic conclusion, more values within the range will replace the base case value. This will be repeated until the exact variable value that changes the study outcome is identified. Reporting this as part of the study results not only indicates the robustness of results, but also boosts the generalizability of them when being interpreted by decision makers in other settings, with different model inputs, e.g., different resource prices.

Uncertainty analysis, by means of Monte Carlo simulation, will be performed via the @Risk-5.0® analysis tool (Palisade Corp., NY, USA) to investigate the likelihood (probability) of a therapy’s economic advantage. Monte Carlo is a method whereby simulated input values, chosen randomly across a range of probability distributions of a model input, are added into the model. The model is run for each simulated input, resulting in a range of outputs characterizing the output uncertainty. Based on this, a distribution of “cost savings” will be generated, indicating the probability of one management option to have an economic advantage over another.

### Ethical considerations and confidentiality

The study protocol and all associated measures, consent forms and recruitment procedures obtained ethical approval by Qatar University (QU) and Hamad Medical Corp. (HMC) institutional review boards (QU-IRB 76/11 and HMC 11113/11). All the participants’ folders are stored in a password-protected database on the site laptop. Only the study pharmacist can access these folders. In addition, all data are retained in a password-protected database maintained along with all related study documentation in a locked cabinet at QU College of Pharmacy.

### Outcome measures

The study research assistants will call the study participants in the intervention and control groups to complete the assessment. To avoid any bias in data collection, the research assistants will be blinded to the participant group allocation.

They will measure the following outcome variables at 3, 6 and 12 months after the start of the study (i.e., the time of the participant’s enrollment in the study):

- Self-reported 7-day point prevalence abstinence, defined as having smoked no cigarettes for the previous 7 days at 3, 6 and 12 months
- Self-reported 30-day point prevalence abstinence defined as having smoked no cigarettes in the last 30 days at 3, 6 and 12 months
- Self-reported continuous abstinence defined as having smoked no cigarettes since quit day at 3, 6 and 12 months
- Smoking abstinence as objectively verified by the CO exhaled test at 12 months
- Mean change in health-related quality-of-life score from baseline at 3, 6 and 12 months for quitters vs. non-quitters
- Overall costs of interventions as studied, including those of utilization for individual recourses consumed
- Average and incremental cost-effectiveness ratio of the study smoking cessation intervention

To objectively determine long-term abstinence, at 12 months, participants who will self-report not smoking for the previous 7 days will be invited to come to their study clinic to measure their exhaled CO level. The clinic nurse who is responsible for measuring the exhaled CO level will be blinded to the study participant group. Participants will be considered abstinent if they register less than 6 ppm on the test. Interpretation of the CO test results will be made carefully taking into consideration time since last smoke, time of the day the test is done and other factors.

Exhaled carbon monoxide is a well-known indicator of cigarette smoking. But because of its short half-life (5-6 h), it only indicates very recent smoking exposure or abstinence. On the other hand, as a result of its relatively long half-life, cotinine can detect nicotine exposure for up to 1 week. However, the use of any NRT formulation can trigger a false-positive urinary cotinine test. Therefore, as it is not expensive and is noninvasive, and it gives the results in a timely manner, the exhaled carbon monoxide test is commonly used in most smoking cessation clinical trials [47].

### Data analysis

The CONSORT guidelines will be followed when analyzing the study data. Demographics and other variables related to smoking and quality of life will be summarized using frequency distributions (for categorical variables) and means with standard deviations (for numeric variables) and will be compared between the two study arms using the chi-squared test (or alternatively the Fisher’s exact test for small cell counts) for categorical variables and the independent *t*-test (or alternatively the Wilcoxon rank sum test for non-normally distributed variables) for numerical variables.

The main outcome will be continuous abstinence at 12 months as self-reported and verified by the CO test. The percentages of participants achieving the main outcome will be computed and compared between the two groups using the chi-squared test. Moreover, the difference in the proportions of continuous abstinence at 12 months between the two study arms (with 95% confidence intervals) will be computed along with the number needed to treat. Multiple logistic regression will be used to obtain the odds ratio of the main outcome between the two study arms adjusting for any imbalances in demographics or other baseline smoking-related variables. Moreover, mixed effects logistic regression, with pharmacy-level random effect, will be considered to adjust for the possible pharmacy effect if it existed. The intent-to-treat principal will be used in the analysis whereby participants who will be lost to follow-up will be classified as smokers and included in the main analysis. This includes those who were removed from the study because they could not tolerate NRT side effects. A sensitivity analysis following that of Borelli, Hogan et al. will be done where the outcome has three possible categories: abstinence, no abstinence and dropout [48]. The suggested multinomial model will allow assessing factors affecting abstinence as well as factors affecting dropout. This will aid in understanding who might need more support during the intervention. Similar analyses for the primary outcome will be done at 3 and 6 months (but do not include verification of abstinence using CO tests).

To assess the participants’ health-related quality of life, SCQOL (smoking cessation quality of life) ratings for each question will be translated into scores. A total health-related quality of life score and a domain-specific score will be calculated at baseline, 3 months, 6 months and 12 months for each participant in the intervention and control groups. The change in the total and domain-specific quality-of-life score from baseline will be calculated for each participant at 3, 6 and 12 months and analyzed using linear mixed models, which will include the quitting (quitters and non-quitters) factor and time factor (at 3, 6 and 12 months) and their interaction.

There is no interim analysis planned for this trial. Statistical analyses will be conducted using the Statistical Package of Social Sciences (IBM-SPSS®), version 21. In all analyses, the level of significance will be set at 5%.

### Sample size calculation

For a priori sample size calculation, we assumed a 7-day point prevalence abstinence at 12 months of 3% for the control group and 15% for the intervention group based on the results of one of the previous studies that assessed the effect of a pharmacist-run smoking cessation program [12]. With a two-sided alpha of 5% and 90% power, a minimum sample size of 118 participants was estimated for each group. This sample size was not adjusted for the potential correlation between outcomes of patients within each clinic since this is not a clustered randomized clinical trial, but in this study patients within each clinic are randomized to the two intervention groups, and correlations, if they exist, will be very small; their effect on the power of the study is negligible [15]. The project has a total of 17 study pharmacists. Each study site is expected to enroll at least 30 participants (15 participants in the intervention group and 15 participants in the control group). The recruitment period is over 12 months (48 weeks). Recruiting at least 30 participants at each study site is considered feasible given that at least 100 patients visit each pharmacy per day. Assuming each study site will recruit 1 participant every week, the 30 needed participants will be recruited in 30 weeks (approximately 8 months). An extra 16 weeks of recruitment time is given to study pharmacists to account for workload, holidays and sickness.

## Discussion

This study is the first randomized controlled trial conducted within Qatar and the Middle East (ME) to test the effect of a pharmacist-delivered smoking cessation program on smoking cessation rates. Furthermore, it is the first study in Qatar to assess the change in health-related quality of life after quitting smoking.

Having the economic analysis component in this study is also of unique significance. To our knowledge, there are no economic studies that have evaluated the economics of a smoking cessation program in Qatar or the region. This component is important as in settings where resources are scarce, proving that an intervention brings a good return to the investment can be as important as proving the intervention effective.

Indeed, in consideration of limited resources and tight departmental budgets, the additional spending associated with any of the study options should be examined. This will be performed through determining the management option that yields benefits that are associated with most health costs savings. For example, the structured program in this study is expected to have a favorable comparative effect profile. But if the value that is associated with these advantages is not high enough, having the structured program in settings may not be the priority practice to implement. Here, the current study provides a great opportunity for such analysis, seeing that randomized controlled trials are the ideal source of data (i.e., highest level evidence) to be used in the construction of economic analyses.

This study will be of significance to Qatar, the ME and to some extent the world. It will advance knowledge about the role that the ambulatory pharmacists in Qatar and possibly in the ME can play in smoking cessation. It will show to what extent these pharmacists would be effective in helping smokers quit smoking. This would be important for Qatar and the ME given the high number of cigarette smokers in these countries [49]. This study will also help in comparing the effect of pharmacist-delivered smoking cessation programs in Qatar with the effect of pharmacist-delivered smoking cessation programs in other countries. Many previous studies conducted in Australia, Europe and the USA have demonstrated the effectiveness of pharmacist-run smoking cessation programs [12-20]. Extrapolating the results of these studies to Qatar and the ME is not readily applicable. The pharmacy practice environment in these countries differs from that in Qatar. The pace of improvement in ambulatory pharmacy practice in Qatar, as other ME countries, is slow. Except for a few cognitive and patient-oriented activities, ambulatory pharmacists’ job is limited to prescription processing and medication dispensing [50]. Nevertheless, Qatar’s pharmacists are increasingly interested in defining a well-recognized vocation for themselves in today’s healthcare system and in moving their focus from pharmaceutical products to patients. This study will be of potential significance to ambulatory pharmacists in Qatar. It will put them in a broader perspective than the product-dispensing context. It will promote their role as public health educators and accessible sources of health information. Ambulatory pharmacists are an essential part of the healthcare system in Qatar and are an existing workforce that needs to be given the opportunity to contribute to patient care. By direct involvement of these pharmacists in smoking cessation, this study will help in improving their professional role and in shifting it to a more patient-centered focus and will assist them in having better integration into the healthcare team in Qatar.

## Trial status

Patient recruitment is currently ongoing.

## Abbreviations

WHO: World Health Organization
QoL: Quality of life
FIP: International Pharmaceutical Federation
ASHP: American Society of Health-System Pharmacists
UREP: Undergraduate Research Experience Program
HMC: Hamad Medical Corporation (HMC)
QU: Qatar University
CPP: Clinical Pharmacy and Practice
CPPD: Continuing professional pharmacy development
NRT: Nicotine replacement therapy
CO: Carbon monoxide
FTND: Fagerstrom test for nicotine dependence
SCQOL: Smoking cessation quality of life
SPSS: Statistical Package of Social Sciences
ME: Middle East
